# Supplementary material for: In situ imaging of the atomic phase transition dynamics in metal halide perovskites
Source: Nat Commun. 2023 Nov 6;14:7142. doi: 10.1038/s41467-023-42999-5 (PMC10628210; doi:10.1038/s41467-023-42999-5)
Supplement: Supplementary file 1 — Supplementary Information [file 41467_2023_42999_MOESM1_ESM.pdf]

Supplementary Information

In situ Imaging of the Atomic Phase Transition Dynamics in Metal Halide Perovskites

Mengmeng Ma[1,2], Xuliang Zhang[1,2], Xiao Chen[3], Hao Xiong[3], Liang Xu[1,2], Tao Cheng[1,2], Jianyu Yuan[1,2], Fei Wei[3], Boyuan Shen[1,2]*

[1] Institute of Functional Nano & Soft Materials (FUNSOM), Soochow University, Suzhou, 215123, Jiangsu, PR China.
[2] Jiangsu Key Laboratory of Advanced Negative Carbon Technologies, Soochow University, Suzhou, 215123, Jiangsu, PR China
[3] Beijing Key Laboratory of Green Chemical Reaction Engineering and Technology, Department of Chemical Engineering, Tsinghua University, Beijing 100084, PR China.

*Corresponding author. Email: byshen@suda.edu.cn (B.S.)

Supplementary Fig. 1-15

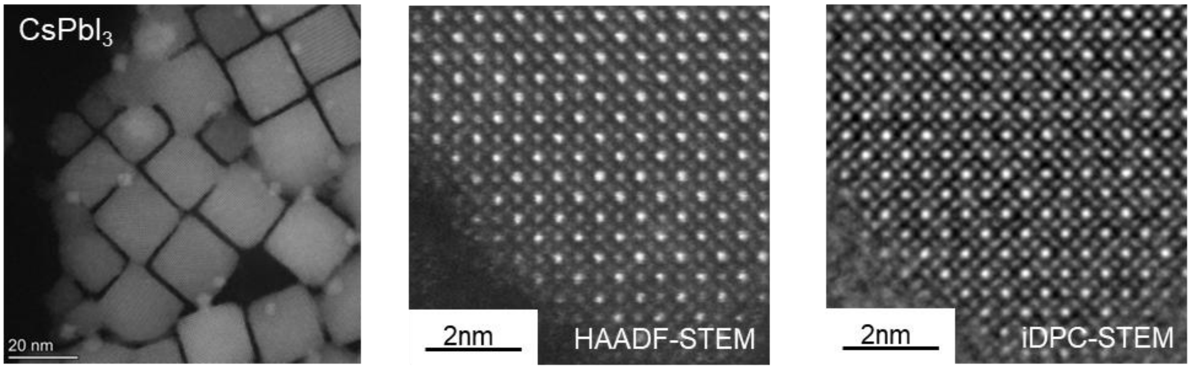

Supplementary Fig. 1 | Comparison of HAADF- and iDPC-STEM images of the same region with the same low dose in $CsPbI_3$ nanocrystal. The intensities of light elements (I and Cs) are higher in the iDPC-STEM image to make them observed clearly.

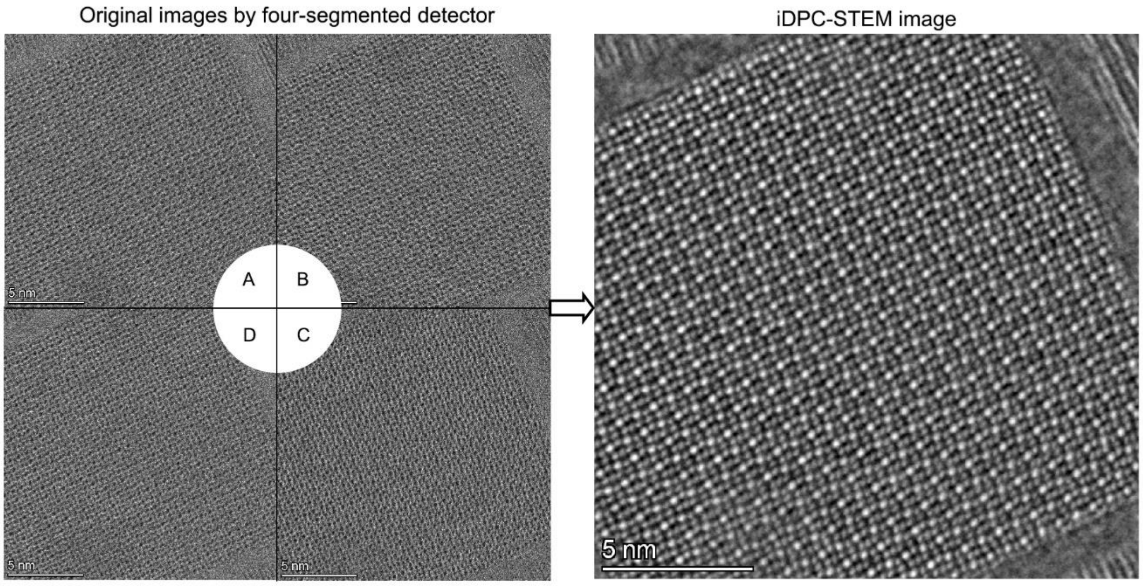

Supplementary Fig. 2 | Process of iDPC-STEM imaging. Four original images (A, B, C and D) were detected by a four-segmented detector. After a 2D integration of the differential image (A-C and B-D), an iDPC-STEM image was obtained with obviously higher resolution.

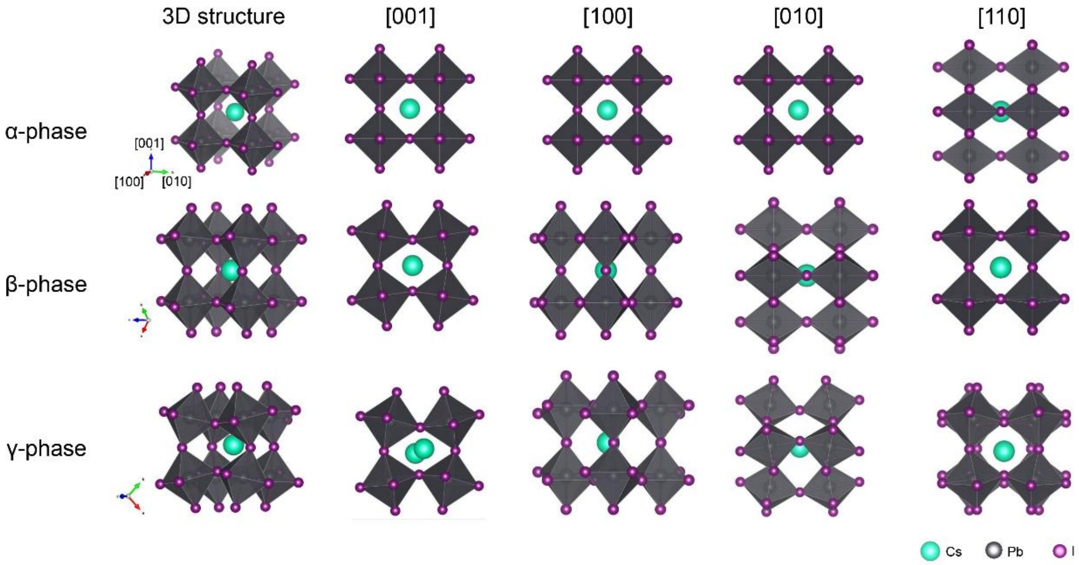

Supplementary Fig. 3 | Structural model of the α, β, and γ phases and viewed from the [001], [100], [010] and [110] directions. The three orthogonal directions of cubic particles correspond to the [001], [110], and [-110] directions of the β and γ phases or the [001], [010], and [100] directions of the α phase, which are defined differently due to their different crystallographic systems (cubic for α, tetragonal for β, and orthorhombic for γ).

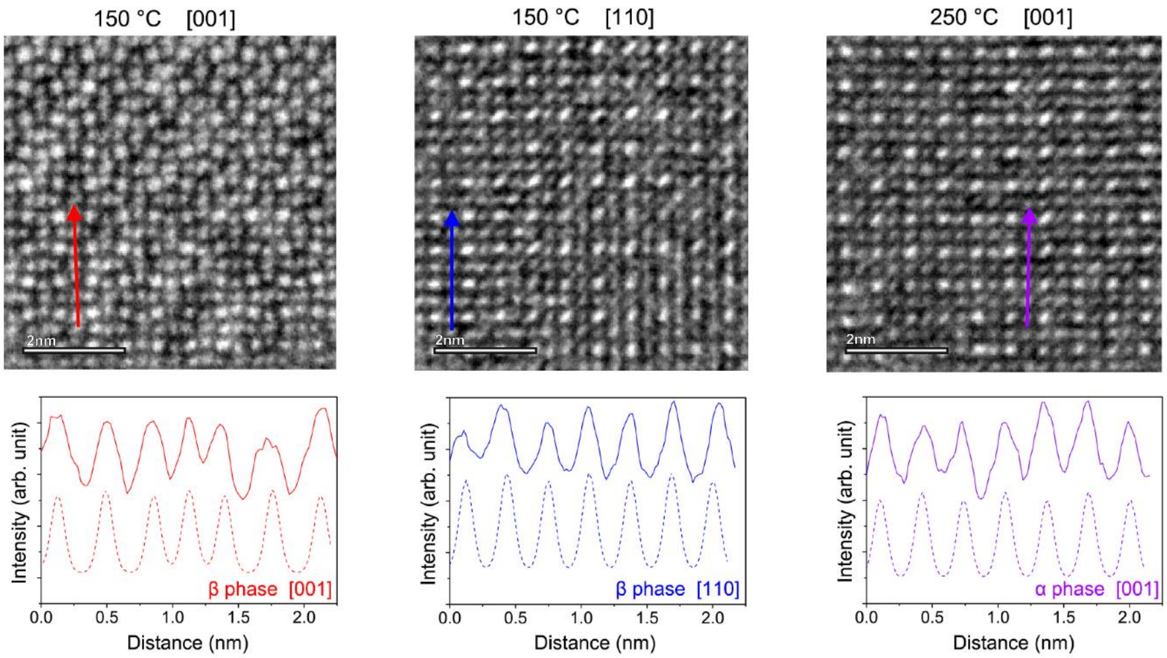

Supplementary Fig. 4 | Supplementary images and profiles during the in-situ heating process. The images and profiles of both the [001] and [110] projections at 150 °C indicate that the

$CsPbI_3$ nanocrystals are in the β phase at 150 °C. We find that the [001] and [110] projections at 250 °C show the same atomic arrangement, which indicates that the $CsPbI_3$ nanocrystals are α phase at 250 °C. They were used to obtain the statistical results in Fig. 2.

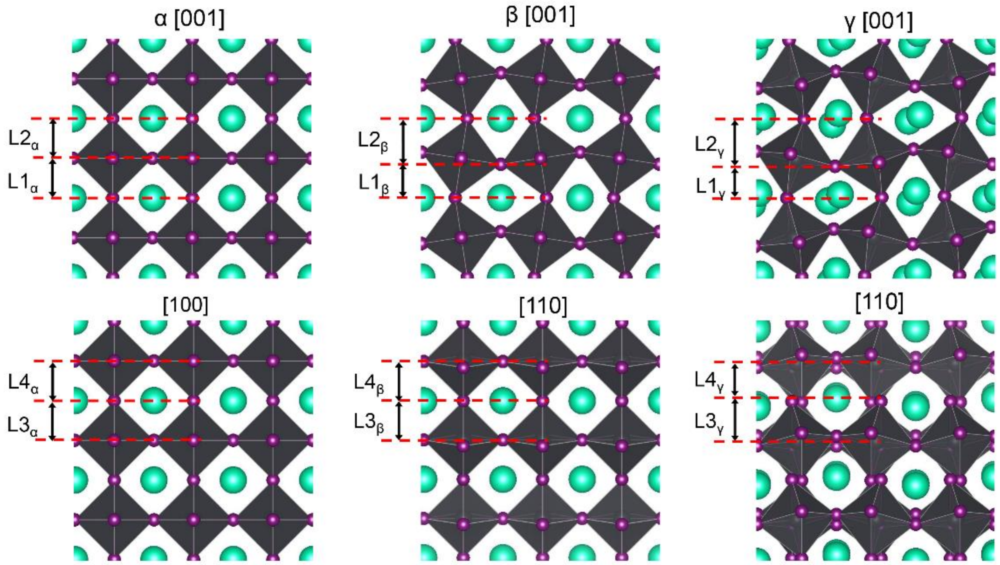

Supplementary Fig. 5 | Schematic diagram of the definition of L1-L4 in Fig. 2. The relations between L1-L4 just indicate the phase of $CsPbI_3$. When changing from γ phase to α phase with temperature, L1 gradually increases to approach L2 in the [001] projection, while L3 gradually decreases to approach L4 in the [110] projection.

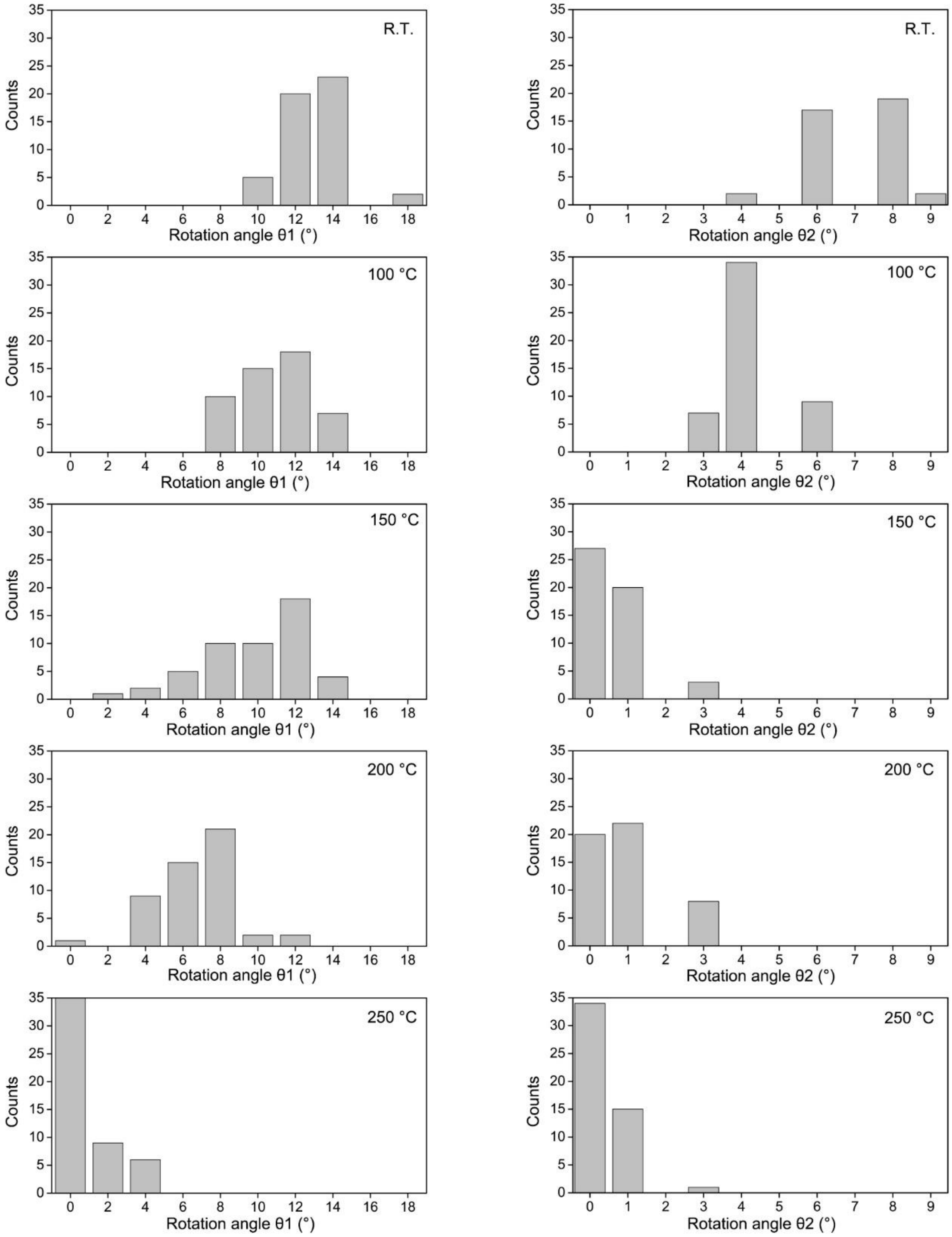

Supplementary Fig. 6 | Distributions of the rotation angles (θ_1 and θ_2) at different temperatures. The rotation angles show a wide distribution at each temperature, which indicates the "intermediate state" during the phase transition process.

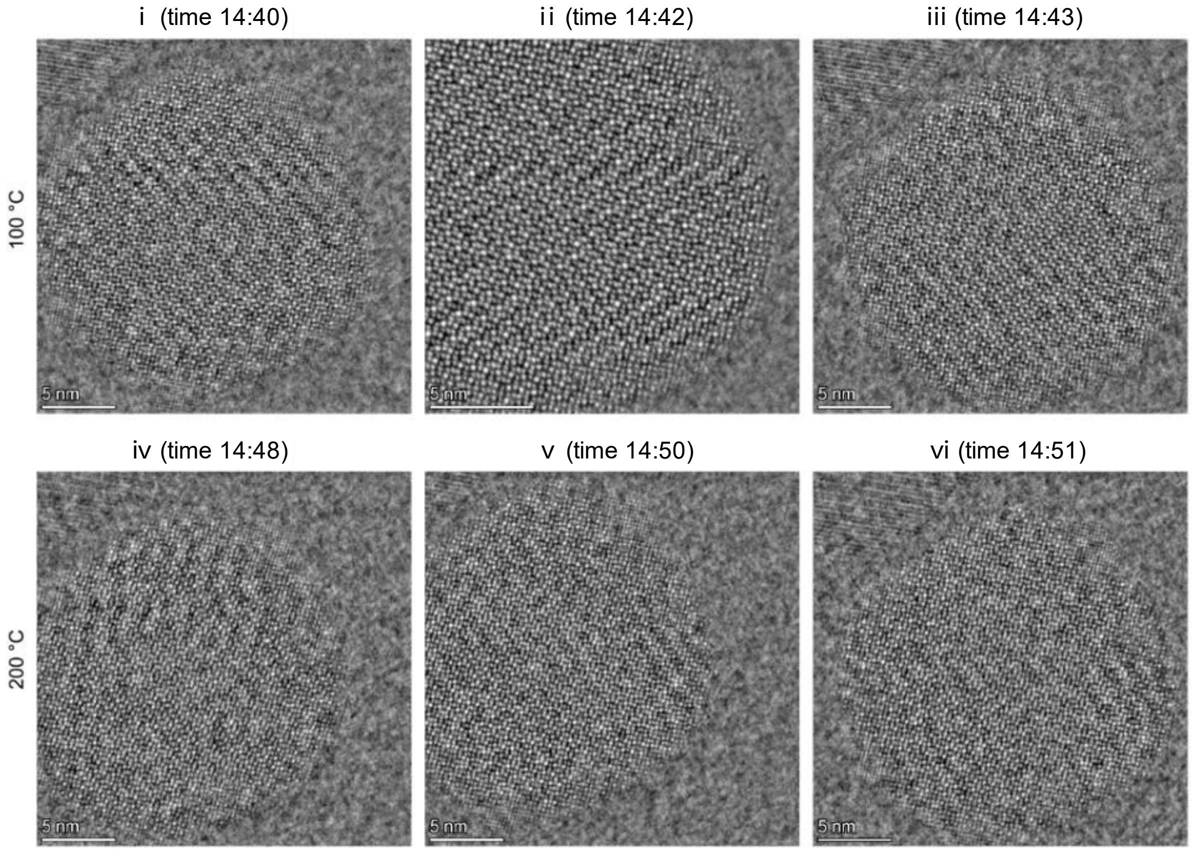

Supplementary Fig. 7 | IDPC-STEM images captured continuously in single $CsPbI_3$ nanocrystal. The figures are numbered from i to vi and marked with the time of image acquisition. Figures i-iii show the phase transition at 100 °C, while figs. iv-vi show the phase transition at 200 °C. At each temperature, the time interval between the image acquisition is approximately one and a half minutes. The actual time stamps are 1.5 min, 3 min, 4.5 min, 9.5 min, 11 min and 12.5 min for these six figures. These figures were used to analyze the phase transition dynamics in Fig. 3.

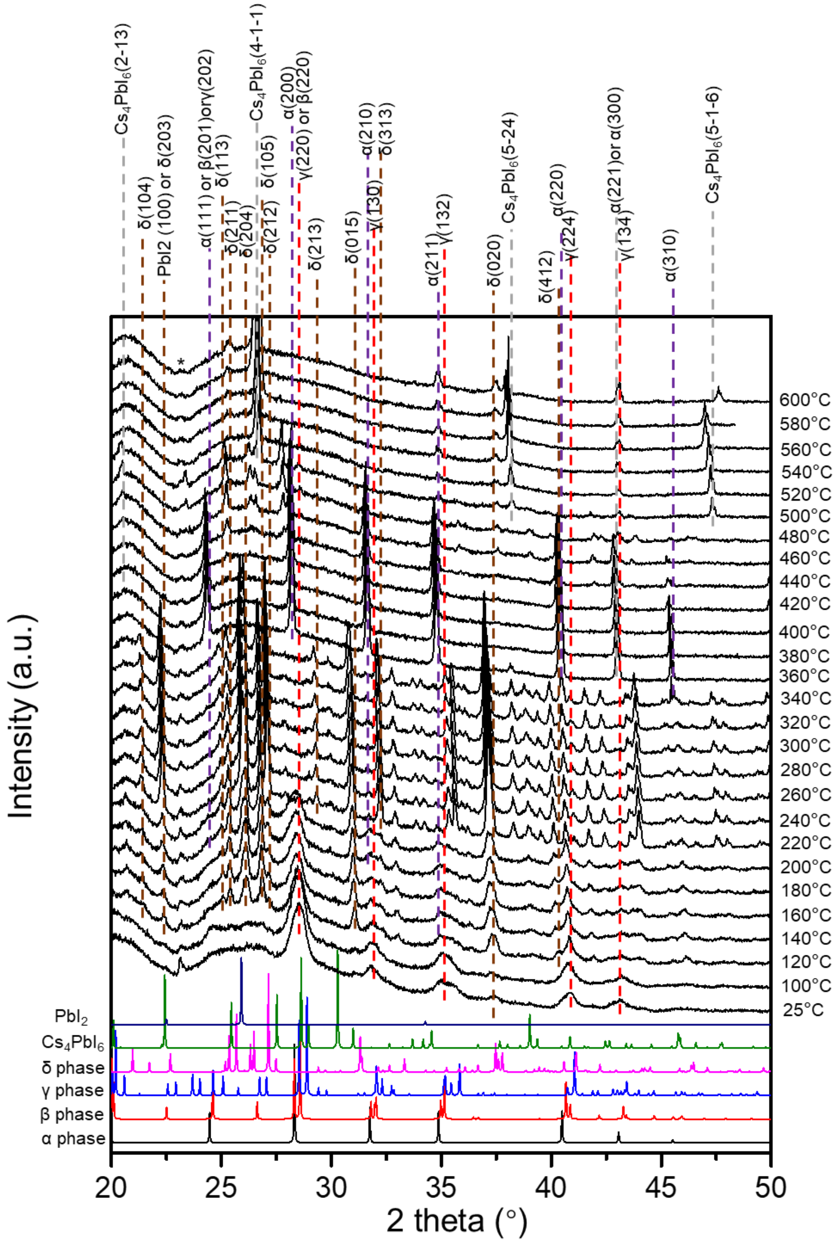

Supplementary Fig. 8 | Analysis of the in-situ XRD results for $CsPbI_3$ nanocrystals. The data are collected at different elevated temperatures ranging from 25 °C to 600 °C and compared with the standard patterns of different phases.

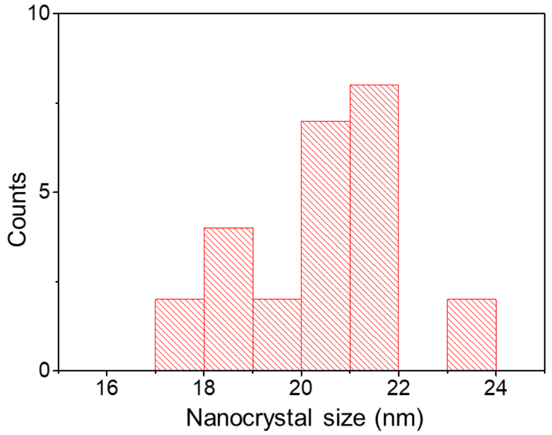

Supplementary Fig. 9 | Histogram of the sizes of nanocrystals in our study. It shows the size of the perovskite crystals is within 10-30 nm.

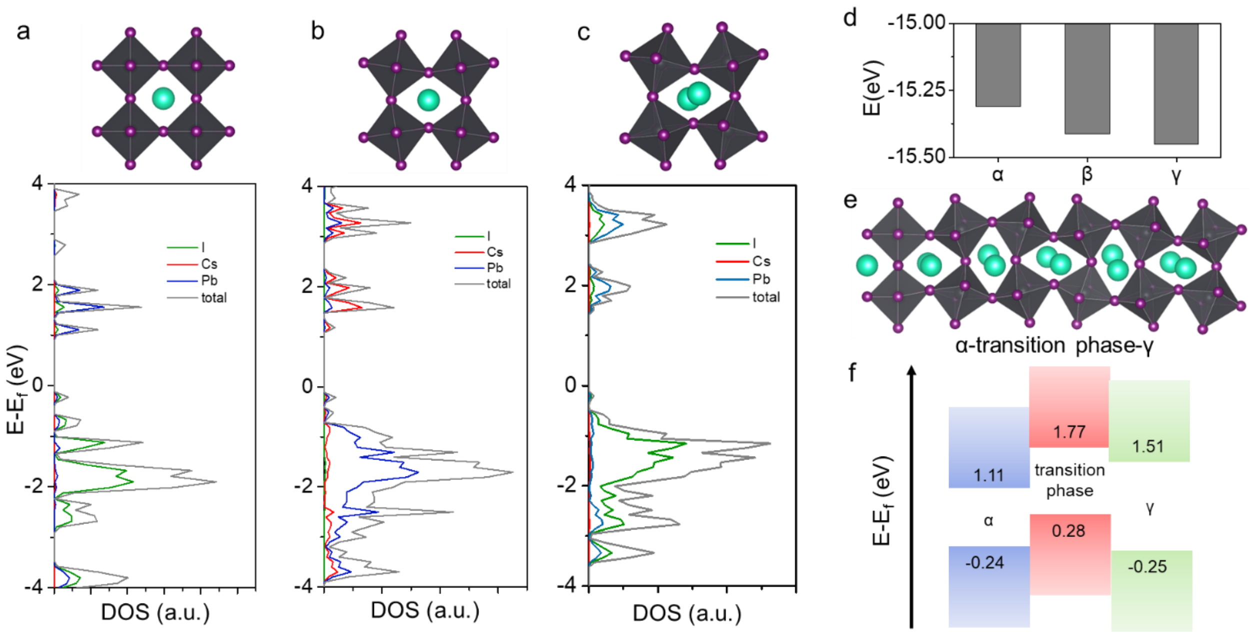

Supplementary Fig. 10 | DFT calculations of $CsPbI_3$ in different phases and transition structure we discussed in Fig. 3. **a-c,** Calculations of DOS in different phases. The band gaps of γ, β and α phase are 1.76, 1.47 and 1.35 eV, respectively. **d**, Calculations of potential energies of different phases ($E_\gamma < E_\beta < E_\alpha$), which shows that the γ phase is the most stable at low temperatures. **e,** Simulated structure of the transition phase between γ and α phase. **f,** Calculated band gap of transition phase, which is closed to the band gap of β phase.

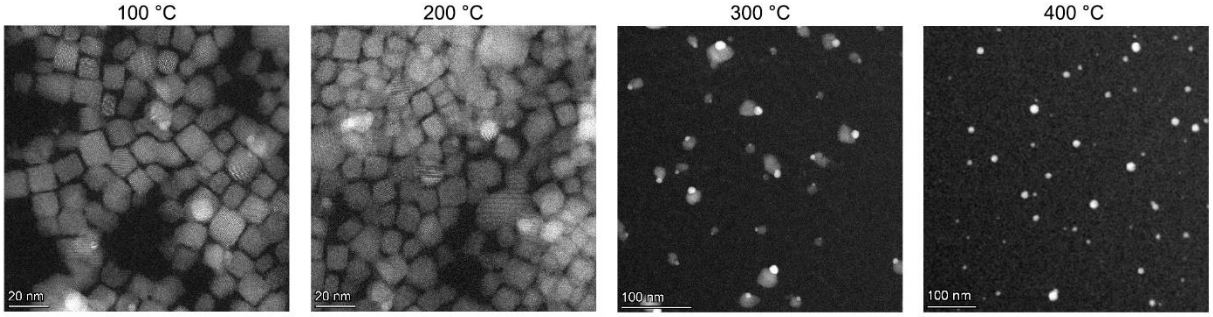

Supplementary Fig. 11 | Supplementary images showing the structural changes of nanocrystals at the particle level. It is clear that the particle morphology will change dramatically at 300 °C.

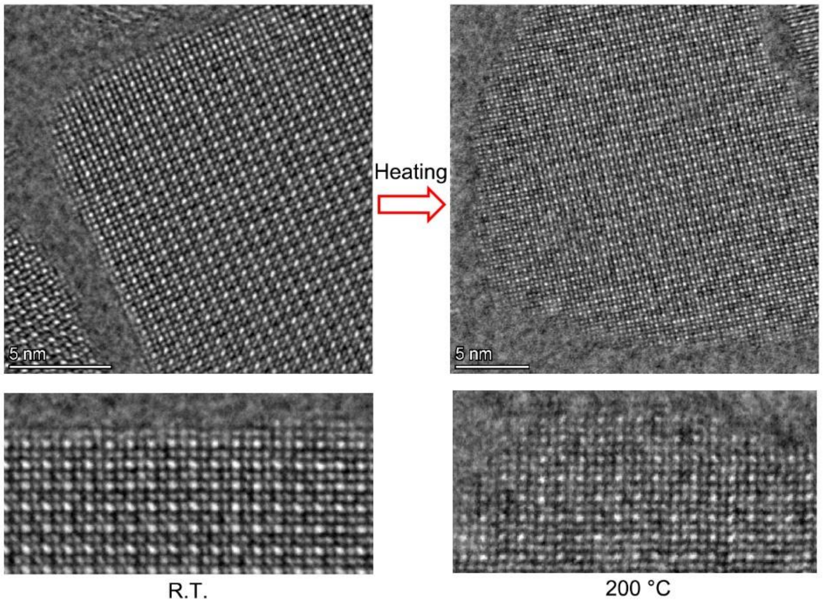

Supplementary Fig. 12 | IDPC-STEM images showing the changes in surface structures with temperature. The flat (001) surface changes into a rugged surface with the change in particle morphology.

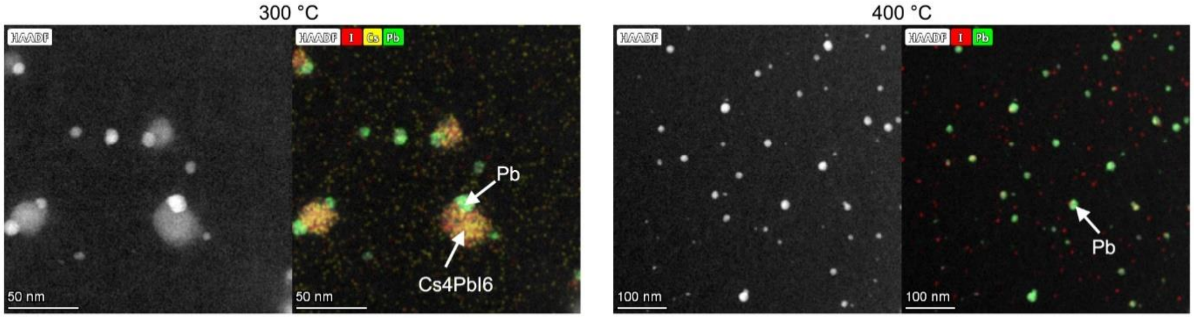

Supplementary Fig. 13 | Elemental mapping of samples at 300 and 400 °C. At 300 °C, Pb and Cs/I are roughly separate indicating that the $CsPbI_3$ nanocrystals were decomposed into Pb and Cs_4PbI_6 particles. At 400 °C, only Pb particles were left.

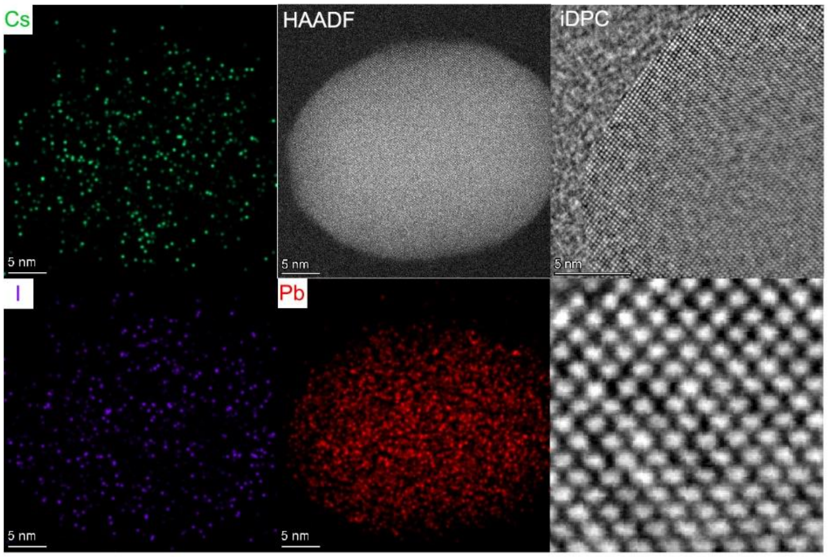

Supplementary Fig. 14 | Elemental mapping and iDPC-STEM images of a Pb particle. The elemental analysis and atomic arrangement confirm the composition and structure of this Pb particle.

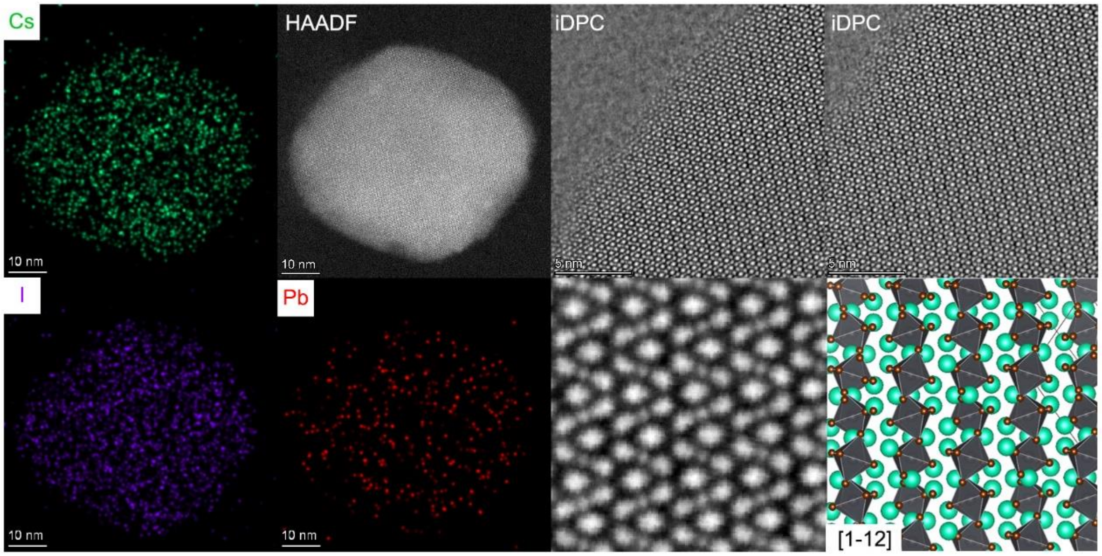

Supplementary Fig. 15 | Elemental mapping and iDPC-STEM images of a Cs_4PbI_6 particle (from another direction). The elemental analysis and atomic arrangement confirm the composition and structure of this Cs_4PbI_6 particle.
